# Large scale patterns in vertical distribution and behaviour of mesopelagic scattering layers

**DOI:** 10.1038/srep19873

**Published:** 2016-01-27

**Authors:** T. A. Klevjer, X. Irigoien, A. Røstad, E. Fraile-Nuez, V. M. Benítez-Barrios, S. Kaartvedt.

**Affiliations:** 1 King Abdullah University of Science and Technology (KAUST), Red Sea Research Center, Thuwal 23955-6900, Saudi Arabia; 2 Institute of Marine Research, PO Box 1870 Nordnes, 5817 Bergen, Norway; 3 Instituto Español de Oceanografía (IEO), Centro Oceanográfico de Canarias, Santa Cruz de Tenerife, E38180, Spain; 4 University of Oslo, Department of Biosciences, PO Box 1066 Blindern, 0316 Oslo, Norway

## Abstract

Recent studies suggest that previous estimates of mesopelagic biomasses are severely biased, with the new, higher estimates underlining the need to unveil behaviourally mediated coupling between shallow and deep ocean habitats. We analysed vertical distribution and diel vertical migration (DVM) of mesopelagic acoustic scattering layers (SLs) recorded at 38 kHz across oceanographic regimes encountered during the circumglobal Malaspina expedition. Mesopelagic SLs were observed in all areas covered, but vertical distributions and DVM patterns varied markedly. The distribution of mesopelagic backscatter was deepest in the southern Indian Ocean (weighted mean daytime depth: WMD 590 m) and shallowest at the oxygen minimum zone in the eastern Pacific (WMD 350 m). DVM was evident in all areas covered, on average ~50% of mesopelagic backscatter made daily excursions from mesopelagic depths to shallow waters. There were marked differences in migrating proportions between the regions, ranging from ~20% in the Indian Ocean to ~90% in the Eastern Pacific. Overall the data suggest strong spatial gradients in mesopelagic DVM patterns, with implied ecological and biogeochemical consequences. Our results suggest that parts of this spatial variability can be explained by horizontal patterns in physical-chemical properties of water masses, such as oxygen, temperature and turbidity.

The gravitational flux of organic particles out of the narrow vertical range where primary production occurs is usually thought to be the main mechanism of the biological carbon pump. The vertical settling speeds are strongly influenced by the size and type of primary producers^1^, and it is also well documented that animal ingestion and repackaging of organic material modify export flux rates^2^. Vertical coupling of the shallow and deep ocean are therefore heavily influenced by ocean ecology. Planktonic consumers frequently migrate vertically, thereby also transporting energy and nutrients, further influencing the vertical coupling of the oceans^3^. Recent studies have suggested that the biomasses of mesopelagic animals, i.e. those animals inhabiting waters from 200–1000 m depth, have previously been severely underestimated^4^,^5^. Since many mesopelagic organisms migrate vertically, this has consequences also for the vertical coupling. Here we use acoustic data from the Malaspina expedition^5^, spanning some of the major oligotrophic oceanic gyres as well as more productive regions at the equator and in the tropics, to elucidate patterns of mesopelagic diel vertical migration behaviour. Since we use single frequency acoustics, our results do not encompass all mesopelagic animals, but are geared towards the larger micronektonic components that are acoustically detectable, particularly mesopelagic fish with a gas-filled swim bladder. We here focus on the patterns of vertical distribution and proportions of acoustic backscatter moving between the deep and shallow ocean on a daily basis.

Diel vertical migration (DVM) has been described as the largest animal movement on earth in terms of biomass^6^. In the typical pattern of DVM organisms reside in deeper waters at day, apparently to avoid visual predators, while foraging at night in upper waters in the shelter of darkness^6^. DVM is mainly performed by organisms inhabiting the two upper vertical “zones” of the ocean, the epipelagic (0–~200 m depth) and the mesopelagic, although also some bathyal organisms perform DVM^7^.The first global biomass estimate of mesopelagic fishes was at 1000 million tonnes^8^, but this value may be an order of magnitude too low^4^,^5^. Long-ranging DVM and high biomass imply that mesopelagic fish are an important part of the biological pump^9^,^10^. While studies have quantified the contribution of mesopelagic micronekton to vertical flux locally^11^,^12^,^13^,^14^, little effort has been made to quantify the role of DVM in global-scale biogeochemical cycling^15^.

A first step to understand the role of vertical migrators as vectors of carbon and nitrogen from the surface to deeper layers, is to obtain a better understanding of the diel vertical migration behaviour. An analysis based on a globally compiled data set of migration depths^15^ found that the depths were correlated with oxygen levels. However, migration depth is only one aspect of DVM behaviour. Another essential parameter in describing DVM behaviour is the fraction of populations taking part in DVM, and for micronektonic organisms this has not been addressed on larger scales. DVM behaviour is usually dynamic^16^, and even within single populations different behaviour may be displayed^17^,^18^, for example related to seasonal environmental variations^19^,^20^. While vertical migrations are ubiquitous in the mesopelagic zone, not all mesopelagic organisms carry out DVM. Even within single species individuals may or may not migrate depending on internal state^21^.

## Mesopelagic Scattering Layers and Diel Variation

**Figure 1:**
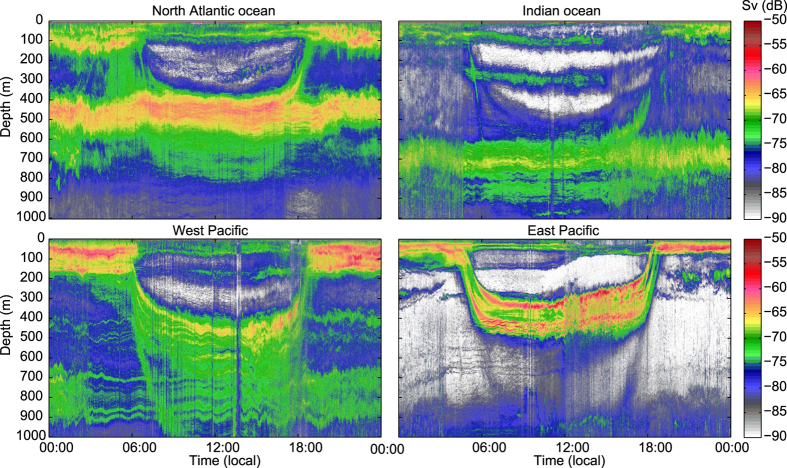
Echograms. Examples of echograms at 38 kHz, spanning 24 hour periods, from different geographic regions, clockwise from upper left: North Atlantic ocean, Indian ocean, East Pacific,West Pacific. Lower threshold is −90 dB.

**Figure 2:**
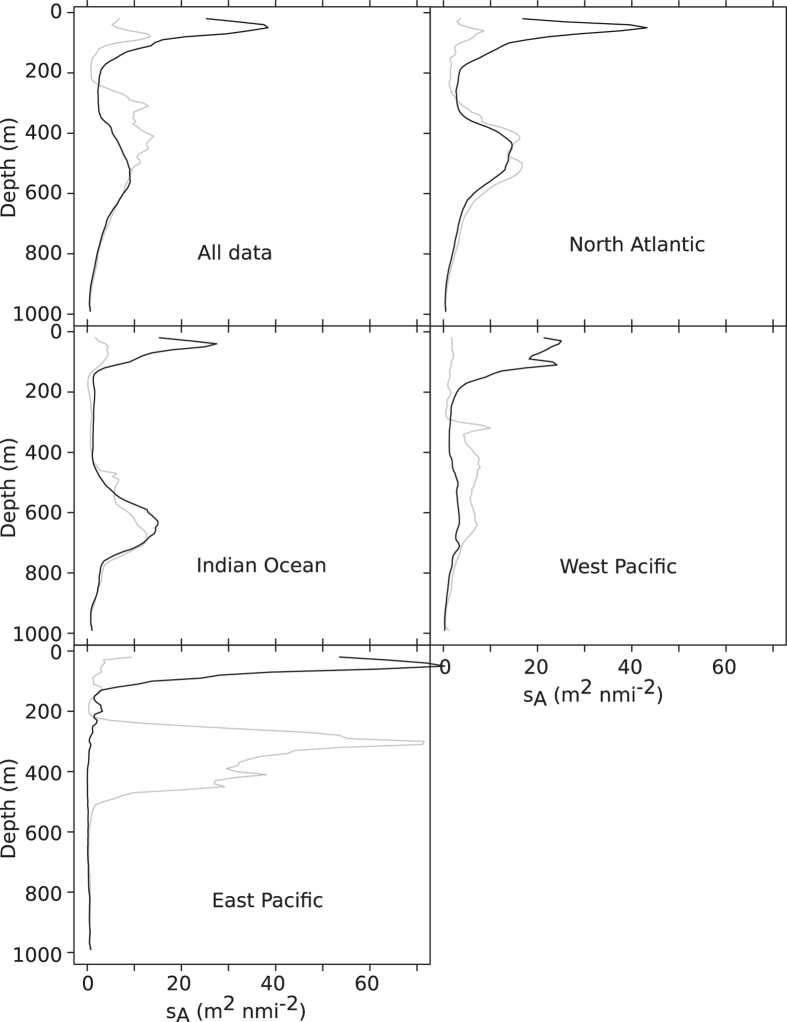
Vertical distributions of backscatter. Vertical distribution of acoustic backscatter grouped into different regions. Black lines show average night-time profiles, gray lines are average daytime profiles. Vertical axis is depth in meters, x-axis are acoustic backscatter measured as NASC per 2 m depth channel (s_A_, m^2^ nmi^−2^).

**Figure 3:**
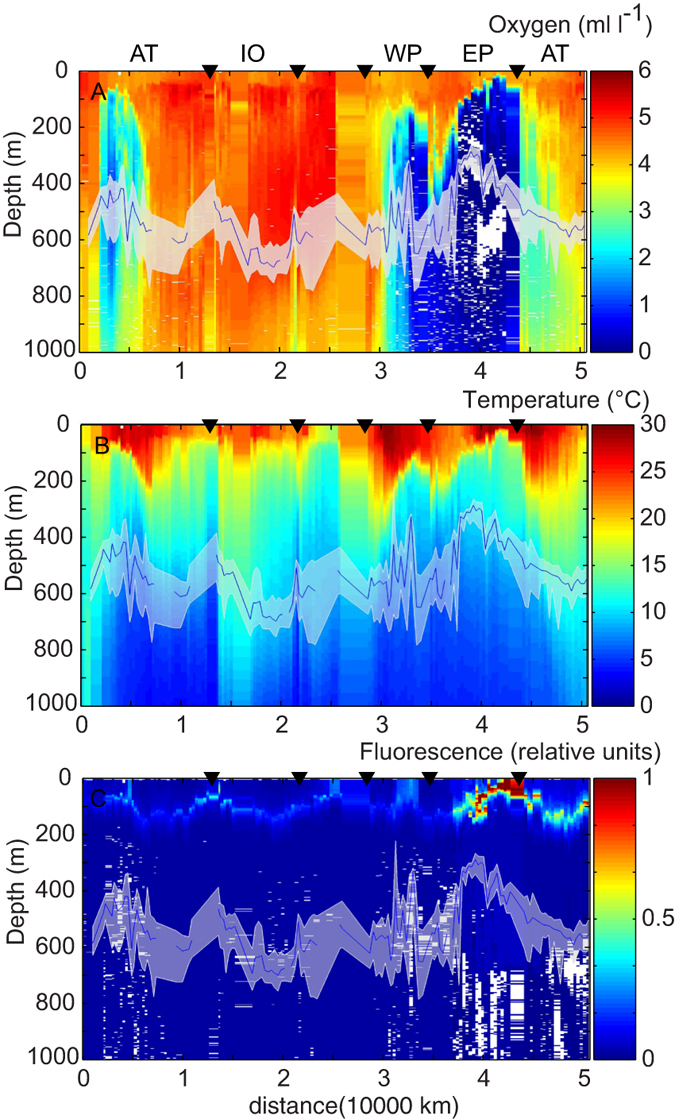
Environment and patterns of behaviour. Median daytime WMD (m, blue line) along with 25 and 75% percentiles of daytime WMD (m) (white band) along the cruise track (km) overlaid on vertical profiles of dissolved oxygen levels (**A**), vertical profiles of temperature (**B**) vertical profiles of fluorescence (**C**). The cruise track is sectioned in different ocean basins by black triangles; starting from left moving throught the Atlantic, Indian Ocean, west Pacific, east Pacific and coming back through the Atlantic ocean.

**Figure 4:**
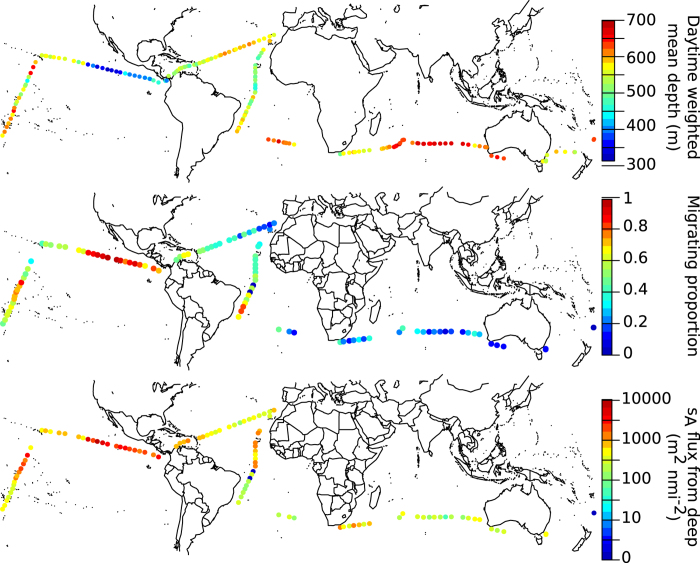
Global patterns in behavior. (**A**) Global map with the cruise track overlayed, showing measured daytime weighted mean depth of acoustic backscatter (WMD, m), as colour. (**B**) Global map with the cruise track overlayed, showing estimated migrating proportion (MP) as colour. Calculation of migration proportion was based on ratio between night and day acoustic backscatter at mesopelagic depths. (**C**) Global map with cruisetrack overlayed, showing estimated net vertical transport from the mesopelagic zone as colour. Net transport was estimated based on change in acoustic backscatter in paired day/night values.

Along the path of the Malaspina cruise daytime scattering layers were found in the mesopelagic zone in all ocean regions (Fig. 1 and 2), but daytime vertical distribution (acoustic weighted mean depth, WMD) varied between the oceans (Table 1, Figs 2,3 and 4A). Table 1 summarizes results for the combined data and also for the different regions.

**Figure 5:**
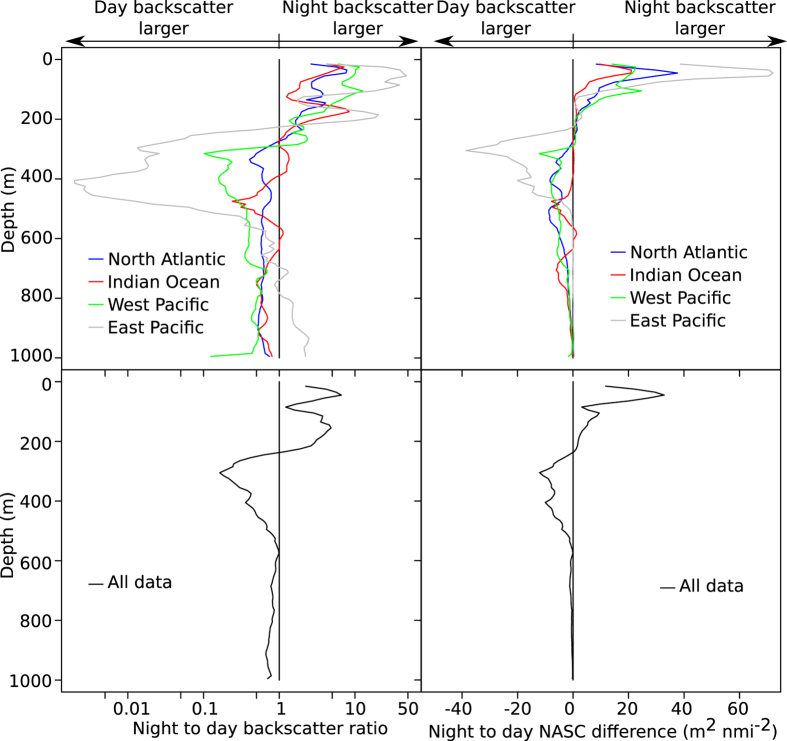
Vertical behavior. Vertical distributions of night to day backscatter ratios (left) and night to day backscatter differences (right) plotted against depth. Note the logarithmic scale in the backscatter ratio plots. The upper panels shows data from different regions indicated by different colours, blue is North Atlantic ocean, red Indian ocean, green Western Pacific ocean, and grey Eastern Pacific ocean. In the lower panels compound measures for cruise total dataset are given. Whereas the plots of backscatter ratio (left panels) reflect to what extent organisms at a certain depth participates in DVM, the plots of backscatter difference (right panels) suggest net backscatter transfer.

In all areas covered by the cruise backscatter decreased at depth and increased in shallow waters (<200 m) during night, as evidenced by the normalized (see methods) ratios of night to day mesopelagic backscatter (Fig. 5). During night acoustic backscatter levels increased above 200 m depths in all regions (Fig. 2), nevertheless, both the North Atlantic and the Indian Ocean datasets also had very clear night-time mesopelagic peaks. For the total dataset the night to day backscatter ratios were below 1 in deep water (i.e. daytime backscatter was larger than night time backscatter at a given depth), and larger than 1 in the epipelagic zone, as expected with normal DVM. In the north Atlantic and the west Pacific the bulk of the migration originated from a relatively wide depth range, ~300–700 m, whereas in the Indian Ocean it appeared to originate from 2 more discrete depth ranges, ~450–500 m and ~650–800 m (Fig. 5).

The data combined for all oceanic areas covered suggested that approximately 50% of the acoustic backscatter remained in the mesopelagic zone during night (Fig. 2 and 5, Table 1). However, there were marked differences in the migrating proportions between the regions, ranging from ~20% in the Indian Ocean to ~90% in the Eastern Pacific (Fig. 2, Table 1). The finer resolution provided by mapping the variables continuously documents variation also within these areas (Fig. 4).

## Influences on Vertical Distribution and Diel Changes

Available environmental variables were checked for correlation with daytime WMD and migrating proportions using a stepwise multiple correlation. Several of the available environmental factors (Table SI 1) were correlated with the WMD. We used average O_2_ 200–1000 m (avOx, measured in ml l^−1^), integrated fluorescence 0–200 m (fluoInt, relative units, a measure of chlorophyll a fluorescence), average turbidity 200–1000 (Turb, relative units, effectively a measure of light scattering), average water column temperature (avTemp, °C), satellite sea surface temperature (sst, °C) and satellite derived chlorophyll a concentrations (chlf, mg m^−3^). Details of parameters and models fitted are given in Supplementary Table SI 1.

For the daytime WMD, average O_2_, integrated fluorescence and average turbidity appeared to explain 77% of the variation (DWMD = 0.89*(avOx) – 0.26*(fluoInt) – 0.17*(Turb) + 2454.7, n = 105, p < 0.001, R^2^ = 0.77). For the migrating proportion the significant explanatory factors were average O_2_, turbidity and sea surface temperature, explaining 72% of the variation (MP = −44*avOx – 15*Turb + 0.52*sst + 0.74, n = 105, p < 0.001, R^2^ = 0.72). We expected differences in behaviour at high and low oxygen levels (see methods). The dataset was therefore split at a threshold of 1.5 ml l^-1^(average O_2_ for 200 to 1000 m).

In low oxygen conditions, oxygen level was the only covariate that was significantly correlated with daytime WMD (R^2^ = 0.75, p ≪ 0.001, N = 21) (Supplementary Fig. SI 1). Nevertheless, in spite of an overall shallower distribution, a large fraction of the backscatter was found deep into the anoxic layer during daytime (Fig. 3). Under high oxygen conditions WMD increased with increasing oxygen levels, and decreased with increasing turbidity, water column temperatures, and both satellite derived chlorophyll and sea surface temperature levels (p ≪ 0.001, N = 81, adj. R^2^ = 0.645)(Supplementary Fig. SI 1).

Under low oxygen conditions (i.e. at concentrations lower than 1.5 ml l^−1^) only water column oxygen and temperature were significantly correlated with the migrating proportion. These factors appeared to explain about 83% of the variation in the migrating proportions (beta regression^22^, n = 21). Oxygen and temperature levels were correlated, and the numbers of observations at low oxygen levels were low. A simplified model without temperature explained about 79% of the variation in the migrating proportions (beta regression, n = 21) (Supplementary Fig. SI 2A).

In high oxygen conditions proportions of migrants increased with increasing sea surface temperatures, but decreased with increasing water column temperatures and turbidities. This altogether explained about 72% of the variation in the data (beta regression, n = 81) (Supplementary Fig. SI 2B,C).

## Diel Vertical Migration

Because of the large areas covered during the Malaspina cruise, the data highlight the large-scale patterns in mesopelagic DVM behaviour (Fig. 1). Recent work refers to the potential importance of DVM by mesopelagic organisms for biogeochemical cycles^13^,^14^,^15^, yet limited information is available for the large scale patterns of biomass and proportion of mesopelagic micronekton actually carrying out DVM. Assessment of vertical fluxes by organisms carrying out DVM cannot be made without that information, and we here provide predictive equations of DVM behaviour for different oceanic regions.

We provide acoustically derived estimates of 2 descriptors of vertical behaviour: the average depth of daytime residence (WMD) and the proportion of animals migrating as indicated by backscatter distribution and changes. Our results suggest that the patterns in these behavioural responses were more uniform within the geographical regions (or stable with time, Fig. 4) than between oceanic areas (Figs 1, 2, 3, 4, 5, Table 1). However, the conclusions (i.e. averages reported in Table 1) are related to the location of the cruise track through the different ocean basins. For example, our results from traversing the southern Indian Ocean suggest a deep vertical distribution and modest vertical migration. These results are superficially similar to what is seen further to the east and somewhat further to the north in the Tasman Sea^23^, but distributions and behaviour are very different in the northern part of the Indian Ocean. Acoustic records in the productive and hypoxic Arabian sea show a shallow distribution^15^, and a high tendency to migration^24^,^25^. In the oligotrophic Red Sea, the daytime distribution is on the other hand deep, but with 95% of the scattering biota migrating towards the surface at night^26^.

Though we present the results as spatial patterns, the expedition spanned one year, with possible seasonal signals in the data. Yet most studies documenting seasonal differences in the mesopelagic are from higher latitudes, where seasonal variations in driving forces are likely more pronounced than in the current study area^18^,^19^. Regardless; the message of considerable variations in DVM behaviour in the largest marine realm (the subtropical and tropical oceans) remains, underlining the need to take such variation into account when assessing the role of dielly migrating mesopelagic organisms for vertical fluxes.

Large-scale geographic variations in daytime mesopelagic scattering layer depths have been known for a long time, and have previously been ascribed to latitude^27^ or variations in light levels^28^,^29^. Recently the depth of migration was linked to oxygen levels, with migration depths shallower in areas with low oxygen levels at midwater depths (OMZ)^15^. Our data confirm that daytime WMDs were inversely correlated with mid water oxygen levels (Fig. 3, Supplementary Fig. SI 1 and SI 2). However, although the correlation of WMD with oxygen is strong, causality remains uncertain. In the areas with anoxia the majority of the backscatter appeared deep into the anoxic layer at day time (Fig. 3), indicating that avoidance of anoxic waters is not the factor resulting in a shallower WMD. One hypothesis is that in the OMZ areas animals may not need to migrate as deep into the ocean interior in order to gain the same level of protection from visual predators with high oxygen demands^15^. Alternatively oxygen might be a proxy for a different factor(s). The WMD reduces the vertical distribution to a single metric, but the importance of forcing factors may vary over the vertical range. For instance, in a recent study from the California current ecosystem it was found that while the lower boundary of the scattering layers correlated with dissolved oxygen, the upper boundary was also correlated with light levels^30^. Establishing the reasons for shallower distributions in areas with midwater OMZ requires further research.

Also the proportion of the community performing DVM was inversely correlated with mid water oxygen levels. Although different mechanisms can help mesopelagic organisms to stay in the hypoxic layers^31^,^32^, oxygen limitation eventually may be critical and can then be offset by vertical migration to the surface during night.

Our data for water column average oxygen values suggest a breakpoint at around 1.5 ml O_2_ l^−1^, at oxygen levels below this the correlation of oxygen with behaviour was very strong (Supplementary Fig. SI 1 and SI 2). The horizontal extent of water masses where the minimum oxygen levels fall below ~0.45 ml l^−1^ concentration (e.g., ~1/3 of the 1.5 ml l^−1^ “breakpoint” in our data, Fig. SI 1 and SI 2) currently makes up 8% of the global ocean surface^33^, with zones of low oxygen expected to expand in the future^34^. While many mesopelagic organisms are adapted to life at low oxygen values^32^, the expansion is likely to result in large-scale changes in mesopelagic behaviour.

In our data, the proportion of the community performing DVM behaviour was strongly correlated to oxygen at low oxygen levels, whereas at higher levels of oxygen other factors were important. The emerging patterns in the diel vertical migration behaviour is the result of a complex set of trade-offs. While low oxygen levels represents a physiological constraint, we lack a clear mechanistic understanding of how the significant factors influenced DVM behaviour. Chlorophyll levels and turbidity may influence *in situ* optical conditions, and temperatures directly influence metabolism and basal rates. However, even without a detailed mechanistic understanding, the high proportion of variance explained in the regression analysis may suggest that simple proxies will improve current models of mesopelagic micronekton behaviour.

## The Migrating Proportion and Vertical Coupling of Shallow and Deep Ocean

The migrating proportion is a key factor since it provides a scaling factor which will affect the active flux component of the biological pump^10^, but it is also a measure of the strength of coupling between the epipelagic and the mesopelagic. We assess the migrating proportion only by using paired day and night values of mesopelagic backscatter (200–1000 m), since we expected organisms from that depth horizon to migrate at least partially into the epipelagic. The implicit assumption is that backscatter lost in the mesopelagic during night is caused by migration out of that depth zone, neglecting potential effects of change in acoustic properties with behaviour and any changes in resonance effects by depth^35^,^36^. We furthermore lack the data to specifically address which mesopelagic components actually performs the DVM, and also to assess how closely the acoustically migrating proportion tracks actual biomass proportions. These are important avenues of future studies.

The total acoustic backscatter involved in DVM from the mesopelagial varies by a factor of 16 between the Eastern Pacific (Estimated migrating NASC, NASC_DVM = 4651 m^2^ n.mi.^−2^) and the Indian Ocean (NASC_DVM = 290 m^2^ n.mi.^−2^), the North Atlantic (NASC_DVM = 830 m^2^ n.mi.^−2^) and West Pacific (NASC_DVM = 1127 m^2^ n.mi.^−2^) have intermediate values. These differences are caused by changes in both absolute backscatter and migrating proportions.

Estimates of the amount of carbon transported out of the epipelagic by interzonal micronektonic migrants have previously been made with models ranging from coarse back-of-the-envelope approximations^9^ to much more detailed models^13^,^14^. Results of the studies vary, with one early estimate^9^ suggesting the possibility of as much as 2.5 mg C m^2^ d^−1^ per 1 g wet weight fish in fecal flux alone, while a more recent study concluded that myctophid contribution to active transport constituted less than 8% of gravitational carbon flux along the North-Atlantic ridge^14^. Another recent modelling study found that the fish mediated export (FME) of carbon in the north-Eastern Pacific nominally was 15–17% (22–24 mg C d^−1^) of total carbon export at shallow depths. However, at a depth of 400 m the flux originating from FME equalled that from passive flux^13^, highlighting the importance of interzonal migrants, repackaging of material and “injection depth” in the biological pump. To some extent our results on spatial variability of DVM patterns may serve to reconcile the discrepancies of these results, as our data suggests that patterns of DVM vary extensively spatially and/or temporally. If the acoustically estimated proportion is a good proxy for the actual biomass proportion participating in DVM, our combined dataset indicate that as much as 50% of mesopelagic micronekton may participate in DVM. Even using an early conservative estimate of mesopelagic fish biomass (1000 million tonnes^5^), this would entail about 500 million tonnes of wet weight moving between shallow waters and mesopelagic depths (e.g 484 m, our large scale average WMD) on a daily basis.

**Figure 6:**
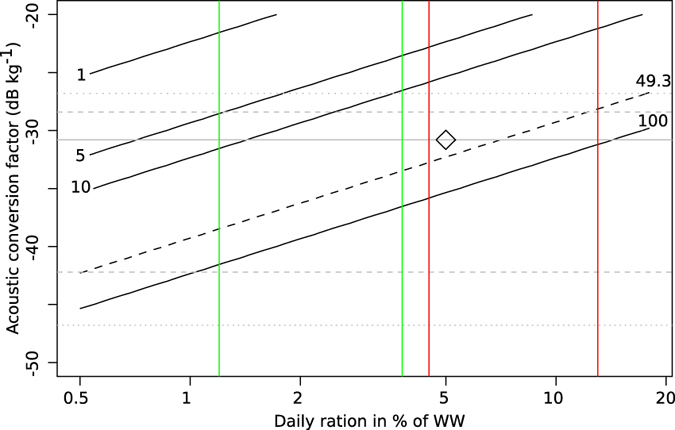
Contour plot of estimated carbon flux in mg C m^−2^ d^−1^ (black lines) as a function of acoustic conversion factor and ingestion, for an acoustic transport of 1000 m^2^ nmi^−2^. The sensitivity model assumes that all ingestion occurs in the epipelagic, that half the ingested carbon is transported to mesopelagic depths, and that the carbon constitutes 5% of the ingested wet weight. The black unbroken diagonal lines are contour lines of estimated carbon flux, delineating flux levels of 1, 5, 10 and 100 mg C m^−2^ d^−1^. The black diagonal dashed line is the contour line of 49.3 mg C m^−2^ d^−1^, corresponding to the global average of gravitational flux out of the epipelagic^37^. Horisontal gray dotted lines are maximum and minimum acoustic conversion values from literature as reported in Irigoien *et al.* 2014^5^, dashed lines are 25 and 75 percentiles, and the grey unbroken line is the median value, all from from Irigoien *et al.* 2014. Green lines indicate range of daily rations reported for myctophids from temperate and subtropical regions^48^ (range 1.2–3.8% of dry body weight, for simplicity we assume that WW to DW ratio is equal in myctophids and prey). Red lines indicate daily rations reported for subtropical and tropical species^48^,^49^ (range 4.5–13% of dry body weight). Using the median acoustic conversion factor from^5^, and a daily ration of 5% of WW, modelled carbon flux would be ~34.9 mg C m^−2^ d^−1^ (black diamond).

To illustrate the potential contribution of micronektonic organisms to vertical carbon flux, we estimated carbon flux in mg C m^−2^ d^−1^ as a function of average acoustic conversion factor and average daily ingestion in % of body weight, for an acoustic migrating flux of 1000 m^2^ nmi^−2^ (Fig. 6), close to our overall cruise average (Table 1). There are many uncertainties in converting acoustic backscatter into carbon and caveats are further outlined in the Method section. Our back-of-the-envelope model assumes that this group forages in the epipelagic, and respires, defecates or otherwise transports half the ingested carbon to mesopelagic depths, and that carbon to wet weight ratio of their prey is 5%^9^. Even considering the uncertainty, the sensitivity analysis (Fig. 6) suggests that the carbon flux driven by mesopelagic fish migration can represent a significant fraction of the total flux, potentially of the same order of magnitude as the gravitational flux^37^. The contribution of the micronektonic mesopelagic vertical migrators to vertical carbon transport is therefore potentially large (Fig. 6), but modelled flux at any given location is very sensitive to changes in input parameters. The actual flux will also depend on a number of other variables, including vertical distribution of feeding, feeding chronology, gut evacuation rates, as well as effects of depth and hydrological conditions on rates and metabolism, among others. Many of these parameters are either presently subject to large uncertainties, or known to vary in space and time, and incorporating them all into models is a difficult task, requiring a level of detail not available to us with the Malaspina data.

The mesopelagic micronekton component is clearly important in the biological pump, and detailed studies^11^,^12^,^13^,^14^ are needed in order to accurately assess its contribution to total flux. Our results show that the importance of mesopelagic micronekton in vertical coupling and carbon transport between the epipelagic and mesopelagic varies between oceanic areas, as has been previously found also for zooplankton^38^,^39^. The combined variability in backscatter and the proportions of diel vertical migrants may introduce variability on the scale of 1 order of magnitude in the amount of carbon transported by mesopelagic micronekton, if biomass per unit backscatter and daily rations are similar between areas. These migrators transport carbon, as well as nutrients, from the epipelagic to mesopelagic depths. Our results suggest that the spatial variability of flux to the deep ocean is increased as a result of variability in mesopelagic behaviour, with the consequence that spatial patterns in the vertical coupling between the deep and shallow ocean is partially under behavioural control.

## Methods

The data presented here are based on the same sampling and were processed in the same manner as the data in Irigoien *et al.*^5^, briefly:

## Echosounder

Acoustic data at 38 kHz were recorded continuously during the cruise (Figs 1,3,4 and 1A in Irigoien *et al.* 2014), using a Simrad EK60 (7° beam width) echosounder calibrated prior to the cruise. Prior to import of acoustic data into the LSSS software^40^, the noisy raw data were filtered using the protocol presented in Irigoien *et al.*^5^,^26^. After manual scrutiny, the remaining data were integrated in 2 minute by 2 meter bins at a threshold of −90 dB in LSSS. The surface exclusion zone varied with weather conditions etc., but were never less than 10 m, densities shallower than this depth were set to the same as in the first depth bin with valid results. The initial data processing for this work deviates from that of Irigoien *et al.* 2014 in that backscattering levels in the 200–1000 m depth horizon were corrected for the effects of the median filtering. This correction was done by correcting binned, integrated backscattering levels at depth according to a smoothed, empirically estimated bias vector (Fig. SI6 in Irigoien *et al.* 2014). Prior to analysis the data were split into day and night data using the R^41^ package “maptools”, according to local sunset/sunrise ± 1 hour.

## Descriptors Used in this Study

We use nautical area scattering coefficients (NASC, s_A_ (m^2^ nmi^−2^)) as a proxy for the acoustically measured biomass. We divided the data into 3 depth-zones: total NASC (s_A-T_, 0–1000 m), epipelagic NASC (s_A-E_, 0–200 m) and mesopelagic NASC (s_A–M_, 200–1000 m). NASC was integrated in channels of 2 meters vertical extension, averaged for a depth-zone, and then multiplied according to zone thicknesses to generate zone NASC.

To assess the vertical distribution of organisms we use weighted mean depth, calculated from the acoustic values:


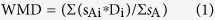


Migration amplitudes were calculated as the difference in WMD between paired day and night observations.

Proportion of animals migrating (MP) was calculated as the ratio between mesopelagic daytime NASC values and mesopelagic nighttime NASC values, corrected for overall NASC changes between day and night (i.e. s_A-T Day_ = s_A-T Night_ after normalisation):


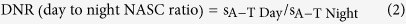



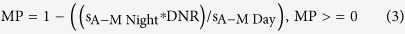


Net transport was calculated as the product of mesopelagic daytime backscatter and migrating proportion, and is interpreted as a measure of diel transport between the mesopelagic and the epipelagic zone.


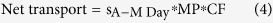


where CF is the acoustic conversion factor, and is 1 for NASC_DVM. Acoustic backscatter increases with increasing densities of organisms^42^. However, the expected conversion factor is a function of both taxonomy and size distributions, both of which may vary spatially. At 38 kHz we expect backscatter from organisms with gas-filled structures to be dominating the backscatter^23^,^43^,^44^. Yet in cases with resonance effects^44^, and reduction in swimbladder air volume with the size of fish, backscatter will not relate reliably with biomass^44^. Also, physonect siphonophores^45^ may bias estimates upwards, while other invertebrates as well as mesopelagic fishes without gas-filled swimbladders^46^ will bias estimates negatively. In a previous paper^5^ we compiled TS results from literature to assess likely ranges for the acoustic conversion factor, we then used summary statistics of the resulting distribution to parametrise biomass estimates. Estimated conversion factors based on these summary statistics are indicated in Fig. 6.

### Additional analysis

We analysed the following variables for correlation with migrating proportion and daytime WMD: Latitude, O_2_ concentration in the water column (average 200–1000 m, average of electronic measurements in units of ml l^−1^), water column temperature (average 200–1000 m in °C), turbidity (average 200–1000 m, measured in relative units), integrated fluorescence (average 0–200 m, relative units), fluorescence at the deep chlorophyll maximum (relative units), sea surface chl a (satellite derived measured in mg chl a m^−3^), sea surface temperature (satellite derived in °C) and attenuation coefficient (satellite derived Kd490 in m^−1^). Low oxygen levels are known to influence vertical behaviour, but when oxygen levels are not limiting, there is little reason to suspect an effect on behaviour. In a large survey of effects of low oxygen on benthic organisms, exposure to concentrations of 1.4 ml l^−1^ appeared to be lethal to 50% of coastal species^47^. Our oxygen levels are based on values averaged over a large vertical range, and are not directly comparable with physiological tolerance levels and point measurements. A preliminary analysis of effects of oxygen levels on behaviour suggested that around a water column average value of 1.5 ml l^−1^ there was a breakpoint in the responses (Supplementary Fig. SI 1 and SI 2).

Based on the distribution of values the measurements of satellite derived chlorophyll a were ln transformed. Latitude and integrated fluorescence were correlated to each other and respectively to oxygen levels and satellite derived sea surface temperature, as well as to oxygen, and these 2 variables were excluded from further analysis. Also the log transformed oxygen levels were correlated with the temperature measures, but were included in the analyses.

For the data sets split according to oxygen conditions, the daytime WMD relationships with environmental variables were then investigated using a stepwise multiple linear regression. The relationships between the migrating proportion and environmental data was analysed using stepwise multiple beta regression in R^41^ (using a logit link, package betareg^22^).

## Additional Information

**How to cite this article**: Klevjer, T. A. *et al.* Large scale patterns in vertical distribution and behaviour of mesopelagic scattering layers. *Sci. Rep.* **6**, 19873; doi: 10.1038/srep19873 (2016).

## Supplementary Material

Supplementary Information

## Figures and Tables

**Table 1:** Summaries of estimated parameters, split according to geographic regions.

	All data combined	Indian Ocean	North Atlantic	East Pacific	West Pacific	Other areas	Unit
Deep daytime NASC (s_A–M_)	2508	1498	2176	5085	1897	2570	m^2^ n.mi.^−2^
Deep daytime S_V_	−71.4	−73.6	−72.0	−68.3	−72.6	−71.3	dB
Deep nighttime NASC (s_A–M_)	1455	1532	1656	233	958	2011	m^2^ n.mi.^−2^
Daytime WMD	486	591	484	348	520	478	m
Nighttime WMD	303	470	319	97	259	331	m
Migration amplitude	183	120	165	251	261	146	m
Migrating proportion (MP)	0.46	0.20	0.38	0.91	0.62	0.35	
Net transport (NASC_DVM)	1156	297	831	4609	1174	910	m^2^ n.mi.^−2^
Day night pairs	105	12	23	12	22	36	

Only datapoints for which paired observations exist are included in the table. Deep refers to mesopelagic (subscript M), i.e. found in the depth range 200–1000 m. WMD refers to weigthed mean depths 0–1000 m (subscript T), WMD = (Σ (s_A–Ti_ * D_i_)/Σ s_A–T_), migration amplitude was calculated as the average day to night difference in WMD. Migrating proportion (MP) was calculated as the ratio between mesopelagic daytime NASC values and mesopelagic nighttime NASC values, corrected for overall NASC changes between day and night (i.e. s_A–T Day_ = s_A–T Night_ after normalisation): DNR = s_A–T Day_/s_A–T Night_, MP = 1− ((s_A–M Night_ * DNR)/s_A–M Day_) , MP > = 0. Net transport was calculated as the average of the product of daytime densities and MP: Net transport = s_A–M Day_ * MP.
