# Supplementary material for: Large scale patterns in vertical distribution and behaviour of mesopelagic scattering layers
Source: Sci Rep. 2016 Jan 27;6:19873. doi: 10.1038/srep19873 (PMC4728495; doi:10.1038/srep19873)
Supplement: Supplementary Information [file srep19873-s1.pdf]

Large scale patterns in vertical distribution and behaviour of mesopelagic scattering layers

Klevjer, T. A.[1,2,*], Irigoien, X.[1], Røstad, A.[1], Fraile-Nuez, E.[3], Benítez-Barrios, V.M.[3], & Kaartvedt. S.[1,4]

Supplementary Information

1. King Abdullah University of Science and Technology (KAUST), Red Sea Research Center, Thuwal 23955-6900, Saudi Arabia
2. Institute of Marine Research, PO Box 1870 Nordnes, 5817 Bergen, Norway.
3. Instituto Español de Oceanografía (IEO), Centro Oceanográfico de Canarias, Santa Cruz de Tenerife, E38180, Spain.
4. University of Oslo, Department of Biosciences, PO Box 1066 Blindern 0316 Oslo, Norway

* Corresponding author: T. A. Klevjer (thor.klevjer@imr.no). Present address Institute of Marine Research, PO Box 1870 Nordnes, 5817 Bergen, Norway.

Supplementary Information:

Model results:
In low oxygen conditions, oxygen level was the only covariate that was significantly correlated with daytime WMD ($R^2 = 0.75$, intercept = 542, beta = 79.64, $p << 0.001$, N=21) (fig. SI 1 A, table SI 1). Under high oxygen conditions WMD increased with increasing oxygen levels (fig. SI 1 A), and decreased with increasing turbidities, water column temperatures, and both satellite derived chlorophyll and sea surface temperature levels. The final model fit (stepwise multiple linear regression) was:

DWMD = 717 + 44*ln(avOx) - 3179*Turb - 7*avTemp - 40*ln(chlf) - 6*sst, $p << 0.001$, N = 81, adj. $R^2 = 0.645$ (High oxygen, $O_2 > 1.5$ ml l^{-1})(fig. SI 1 B, C, table SI 1).

Under low oxygen conditions (i.e. at concentrations lower than 1.5 ml l^{-1}) only water column oxygen and temperature were found to be significantly correlated with migrating proportion, these factors appeared to explain about 83 % of the variation in the migrating proportions (stepwise multiple beta regression[22], estimated intercept 3.354 ($p = 0.0499$), slope ln(avOx) = -1.214 ($p << 0.001$), slope avTemp = -0.476 ($p = 0.045$), $\Phi = 25.593$, n=21, pseudo $R^2 = 0.829$).
Oxygen and temperature levels were correlated, and the numbers of observations at low oxygen levels are low, a simplified model without temperature would explain about 78 % of the variation in the migrating proportions (beta regression, logit link, estimated intercept -0.06 ($p = 0.72$), slope ln(avOx) = -1.095 ($p << 0.001$), $\Phi = 21.201$, n=21, pseudo $R^2 = 0.788$) (fig. SI 2 A, table SI 1).

In high oxygen conditions proportions of migrants increased with increasing sea surface temperatures, but decreased with increasing water column temperatures and turbidities (stepwise multiple beta regression, logit link, estimated intercept= -3.868 ($p << 0.001$), slope sst = 0.237 ($p << 0.001$), slope avTe = -0.17 ($p << 0.001$), slope Turb = -29.526 ($p = 0.0196$), $\Phi = 15.133$, N = 81, pseudo $R^2 = 0.72$) (fig. SI 2 B,C, table SI 1).

Methods:
Postprocessing bias assessment and correction:
Bias introduced through our non-standard post-processing methods were assessed by comparing the results with results from standard acoustic post-processing in low-noise sections, as outlined in [5]. We corrected for vertical bias by adding or subtracting the average bias of that depth to our results, at depths over 200 m (depths < 200 m were not corrected), but truncated high and low bias values (i.e. $-0.5 < \text{bias} < 1$) and smoothed the vertical bias vector with a 20 point running mean:

corrected $s_{AC\,i} = s_{AR\,i} + (B_i * s_{ARi})$, where B_i is the empirically determined bias for depth channel i

Satellite data:. Annual values for chlorophyll a, Kd490 and daytime SST4 data were downloaded directly from http://modis.gsfc.nasa.gov/, and converted to text format using the program swreadl3b (http://0-oceancolor.gsfc.nasa.gov.iii server.ualr.edu/forum/oceancolor/topic_show.pl?pid=3604#pid3604), and imported into R[41]. Data reported in table 1 was compiled based on the CTD stations, i.e. day and night time data within ± 12 hours of the CTD stations were used as matched values.

We split the data into different regions based on geographic location. In the global dataset we include all available data, north Atlantic data is data collected between equator and 40° N and 60° and 20° W. Indian ocean data was collected between latitudes 45° and 25° S and longitudes 50° and 110° E. We split the data for the Pacific ocean, with western Pacific data collected between 180° and 130° W (10° S to 20° N), and eastern Pacific data between 130° W and 90° W (0° to 20° N).

Variable modelled	DWMD	MP	DWMD	DWMD	MP	MP		
Scope	All	All	avOx < 1.5	avOx > 1.5	avOx < 1.5	avOx > 1.5		
Type	Linear	Linear	Linear	Linear	Beta, logit link	Beta, logit link		
N	105	105	21	81	21	81		
p value	<0.001	<0.001	<0.001	<0.001				
R^2/pseudo R^2	0.77	0.72	0.75	0.65	0.83	0.72		
Variables (p value)							Parameter description	Depth range of parameter
Intercept	2454.7	0.74	542	717	3.354 (0.0499)	-3.868 (<0.001)		
avOx	0.89	-44					Average oxygen from CTD casts (ml l-1)	200 – 1000 m
ln(avOx)			79.64	44	-1.214 (<0.001)		Average oxygen from CTD casts (ml l-1)	200 – 1000 m
fluoInt	-0.26						Integrated fluoresence from CTD casts (relative unit)	0 - 200 m
Turb	0.17	15		-3179		-29.526 (0.020)	Average turbidity from CTD casts (relative units)	200 – 1000 m
avTemp				-7	-0.476 (0.045)	-0.17 (<0.001)	Average temperature from CTD casts (°C)	200 – 1000 m
sst		0.52		-6		0.237 (<0.001)	Satellite measured sea surface temperature (°C)	Surface
ln(chlf)				-40			Satellite measured sea chlorophyll levels (mg m^{-3})	Surface
Other info:					$\Phi = 25.593$	$\Phi = 15.133$		
Variables not significant or dropped								
Latitude							Latitude	
Maximum fluorescence level							Maximum deep fluorescence level from CTD casts	0 – 200 m
Satellite derived diffuse attenuation coefficient at 490 nm							Satellite derived diffuse attenuation coefficient (m^{-1})	

Table SI 1: Details of fit models, and variables used in models.

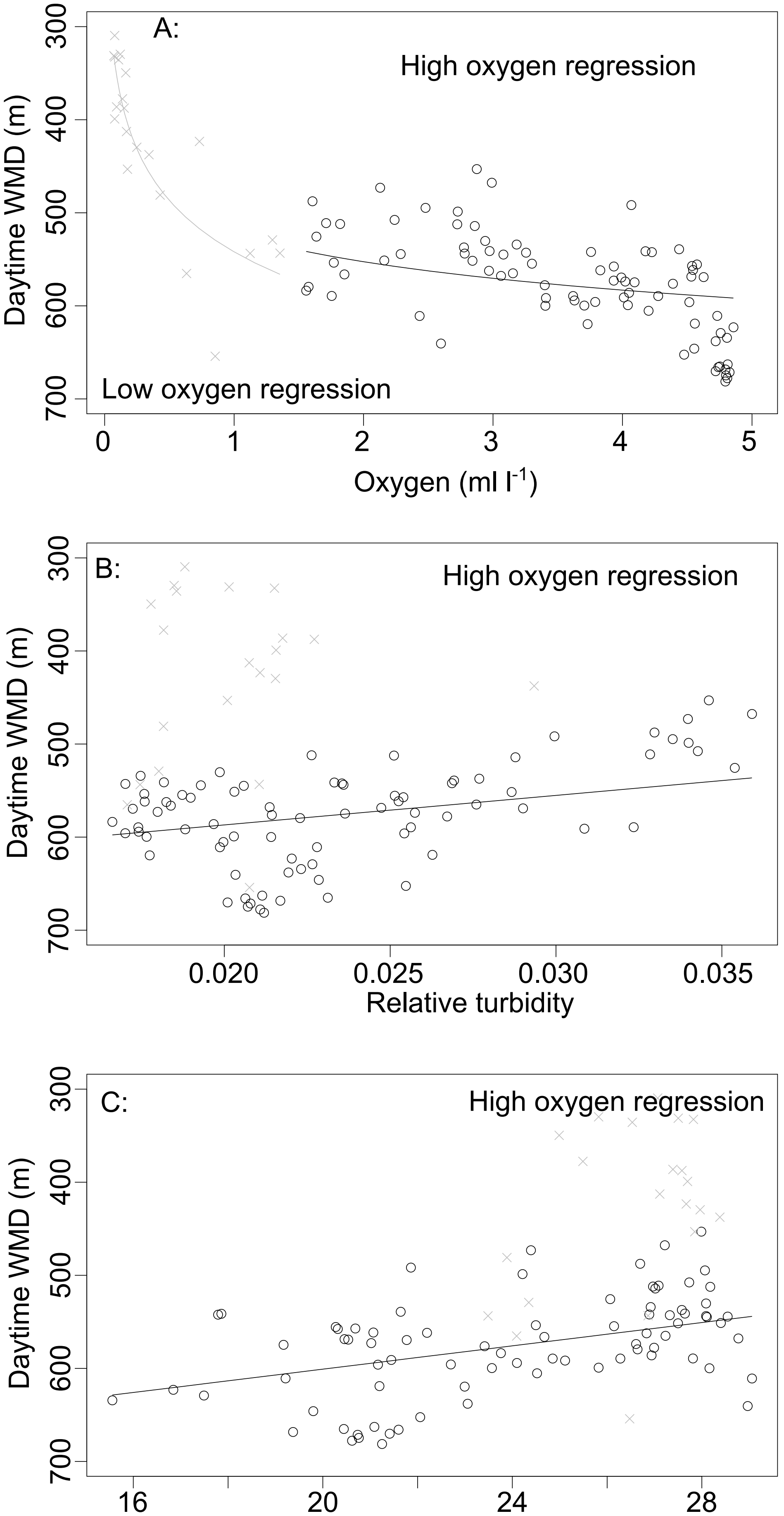
A:
High oxygen regression
Low oxygen regression
Daytime WMD (m)
300
400
500
600
700
0
1
2
3
4
5
Oxygen (ml l-1)
B:
High oxygen regression
Daytime WMD (m)
0.020
0.025
0.030
0.035
Relative turbidity
C:
High oxygen regression
Daytime WMD (m)
16
20
24
28
Sea surface temperature (ºC)

Fig. SI 1: Environment and daytime vertical distribution:

Scatterplots of daytime center of gravity (WMD) against different environmental factors. Squares are oxygen levels below 1.5 ml l^{-1} and circles are oxygen levels above.

A: Daytime center of gravity (WMD) plotted against average water column oxygen values (200-1000m). Grey line shows regression line for low oxygen data and black line show regression for high oxygen data.

B: Daytime WMD plotted against average water column turbidity (relative values, 200-1000m). Black line shows the modelled response of WMD as a function of turbidity, with other significant factors in the multiple regression model set to average values. Only high oxygen data were included in this model.

C: Daytime WMD plotted against average water column temperatures (200-1000m). Black line shows the modelled response of WMD as a function of temperatures, with other significant factors in the multiple regression model set to average values. Only high oxygen data were included in this model

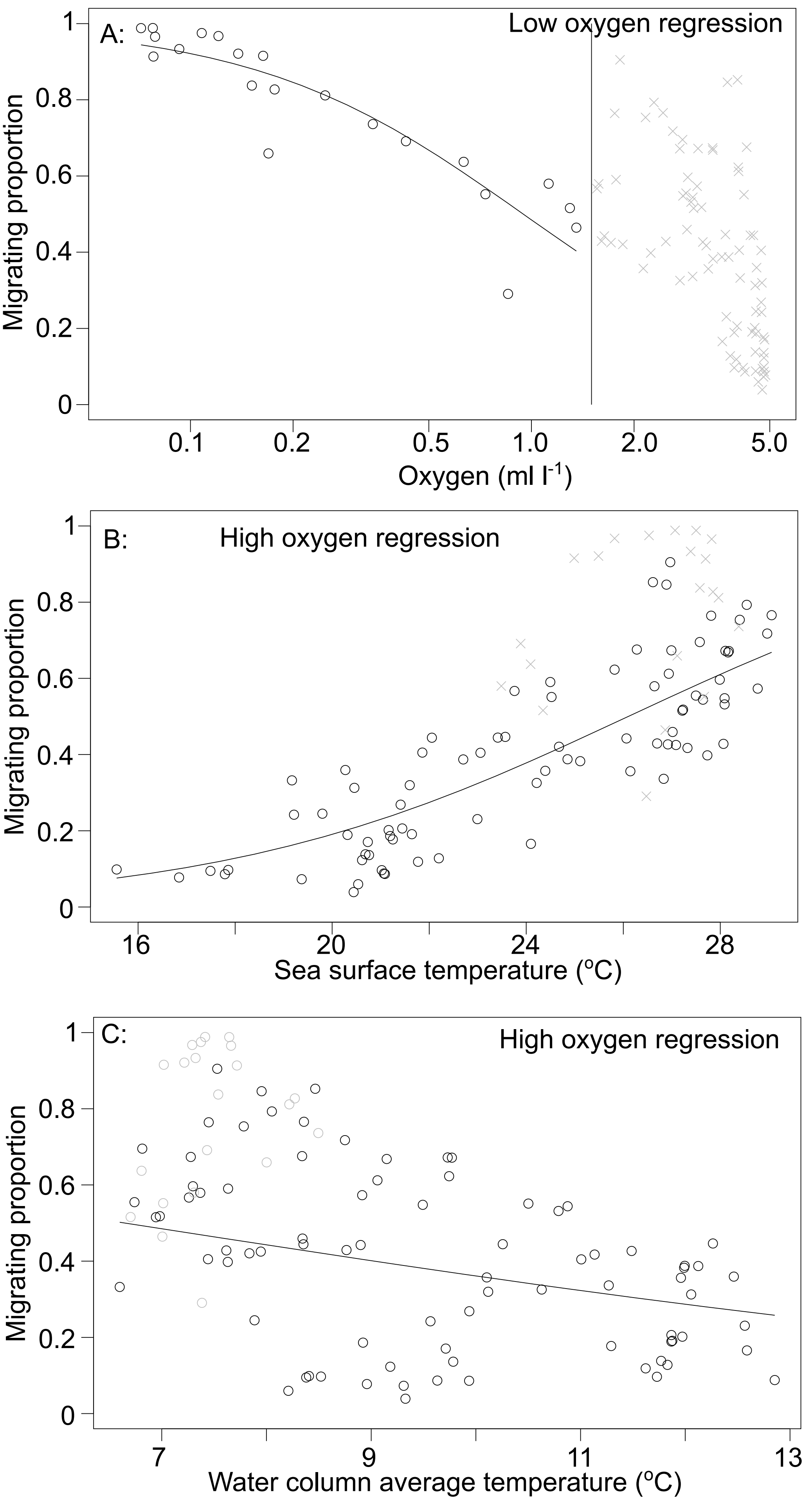

A:
Low oxygen regression
Migrating proportion
Oxygen (ml l-1)
B:
High oxygen regression
Migrating proportion
Sea surface temperature (oC)
C:
High oxygen regression
Migrating proportion
Water column average temperature (oC)

Fig. SI 2: Environment and migration proportion:

Scatterplots of migrating proportions against different environmental factors.

A: Migrating proportion plotted against average water column oxygen values (200-1000m). Black line shows regression line for low oxygen data. Black circles denote oxygen levels below 1.5 ml l^{-1} and gray X symbols denote oxygen levels above this limit.

B: Migrating proportion plotted against satellite derived sea surface temperatures. Black line shows the modelled response of migration proportion as a function of sea surface temperatures, with other significant factors in the multiple regression model set to average values. Black circles denote oxygen levels below 1.5 ml l^{-1} and gray X symbols denote oxygen levels above this limit. Only high oxygen data (black circles) were included in this model

C: Migrating proportion plotted against water column average temperatures (200-1000m). Black line shows the modelled response of migration proportion as a function of water column temperatures, with other significant factors in the multiple regression model set to average values. Black circles denote oxygen levels below 1.5 ml l^{-1} and gray circles denote oxygen levels above this limit. Only high oxygen data (black circles) were included in this model.
